# Epileptic Seizures in a Pediatric Patient With Vein of Galen Aneurysmal Malformation and Obstructive Hydrocephalus: A Rare Case Report

**DOI:** 10.7759/cureus.56962

**Published:** 2024-03-26

**Authors:** Kiril Ivanov, Stanimir Atsev, Petar-Preslav Petrov, Ilko Ilyov, Plamen Penchev

**Affiliations:** 1 Faculty of Medicine, Medical University of Plovdiv, Plovdiv, BGR; 2 Cardiac Surgery Clinic, Passau Clinic, Passau, DEU; 3 Department of Anatomy, Histology and Embryology, Medical University of Plovdiv, Plovdiv, BGR

**Keywords:** surgical case reports, case report, epileptic seizures, endoscopic third ventriculostomy, endovascular embolization, obstructive hydrocephalus, vein of galen aneurysmatic malformation

## Abstract

The vein of Galen aneurysmal malformation (VGAM) is a rare congenital arteriovenous fistula of the embryonic median prosencephalic vein of Markowski, resulting in its pathological dilation. If left untreated, it can lead to multiple severe complications in the neonatal period, among which obstructive hydrocephalus. We present a case report of a six-year-old male patient with severe status epilepticus and a clinical history of VGAM and obstructive hydrocephalus, diagnosed via an MRI and an MR-angiography. The hydrocephalus was treated via a ventriculostomy at the age of six months, while the VGAM underwent a partial transarterial endovascular embolization when the patient was four years old. The procedures were successful, and there were no significant post-operative complications observed. The epileptic seizures began at a later point and were successfully medicated with valproate. However, they resumed due to a lowering of the medication dosage by the patient’s parents. The patient was given a new valproic acid regimen with an appropriate dosage, and his parents reported no further seizures. This case report emphasizes the use of appropriate prenatal and neonatal diagnostic methods for VGAM and explores the nature of the multi-procedural therapy approach towards the pathology and its complications in relation to a possibly idiopathic co-pathology, namely epilepsy.

## Introduction

The vein of Galen aneurysmal malformation (VGAM) is a rare congenital arteriovenous fistula in which one or multiple choroidal and thalamic feeders shunt into the median prosencephalic vein of Markowski and dilate it pathologically, causing complications such as hydrocephalus, congestive heart failure (CHF), and seizures [1-3]. It is a rare abnormality, occurring in one in 25,000 births [2]. The lesion can be diagnosed via antenatal and neonatal imaging, such as MRI, MR-angiography, ultrasound, and Doppler [1,4]. Treatment includes surgery, medication, and endovascular embolization, with transarterial embolization remaining the gold standard in pediatric cases [3,5-7]. Prognosis depends on multiple factors, including the size and type of the lesion, the neurological and physiological status of the patient, proper planning and delivery of the pregnancy based on antenatal diagnosis, and the presence of complications and comorbidities [4,8]. Obstructive hydrocephalus is a common complication in patients with VGAM, presenting with enlarged ventricles and cranium due to the dilated vein of Markowski compressing the aqueduct of Sylvius [1,9].

The preferred treatment method for obstructive hydrocephalus in such cases (when embolization of the VGAM cannot be performed or is insufficient by itself) in neonates and infants is endoscopic third ventriculostomy (ETV), which is associated with less severe and more infrequent long-term complications when compared to the alternative, a ventriculoperitoneal (VP) shunt [5,9-11]. Epilepsy is not a complication associated with VGAM or its treatment methods, although there is a single case report reporting it as secondary to VGAM in an adult patient [1,12,13]. In this report, we present the case of a six-year-old male patient suffering from epileptic seizures after previous treatment for obstructive hydrocephalus via ETV and a VGAM treated via transarterial endovascular embolization. We aim to explore the reliability of the treatment approach for the patient’s congenital abnormalities and their possible correlation with the exhibited epileptic seizures.

## Case presentation

A six-year-old male has presented to the ER after a severe episode of convulsive status epilepticus with tonic-clonic seizures. The child had a clinical history of seizures and had been diagnosed with epilepsy for nearly two years, alongside a rare combination of previously treated congenital comorbidities, VGAM, and obstructive hydrocephalus. The patient had normal intellectual development and no neurological deficits reported up to that point.

**Figure 1:**
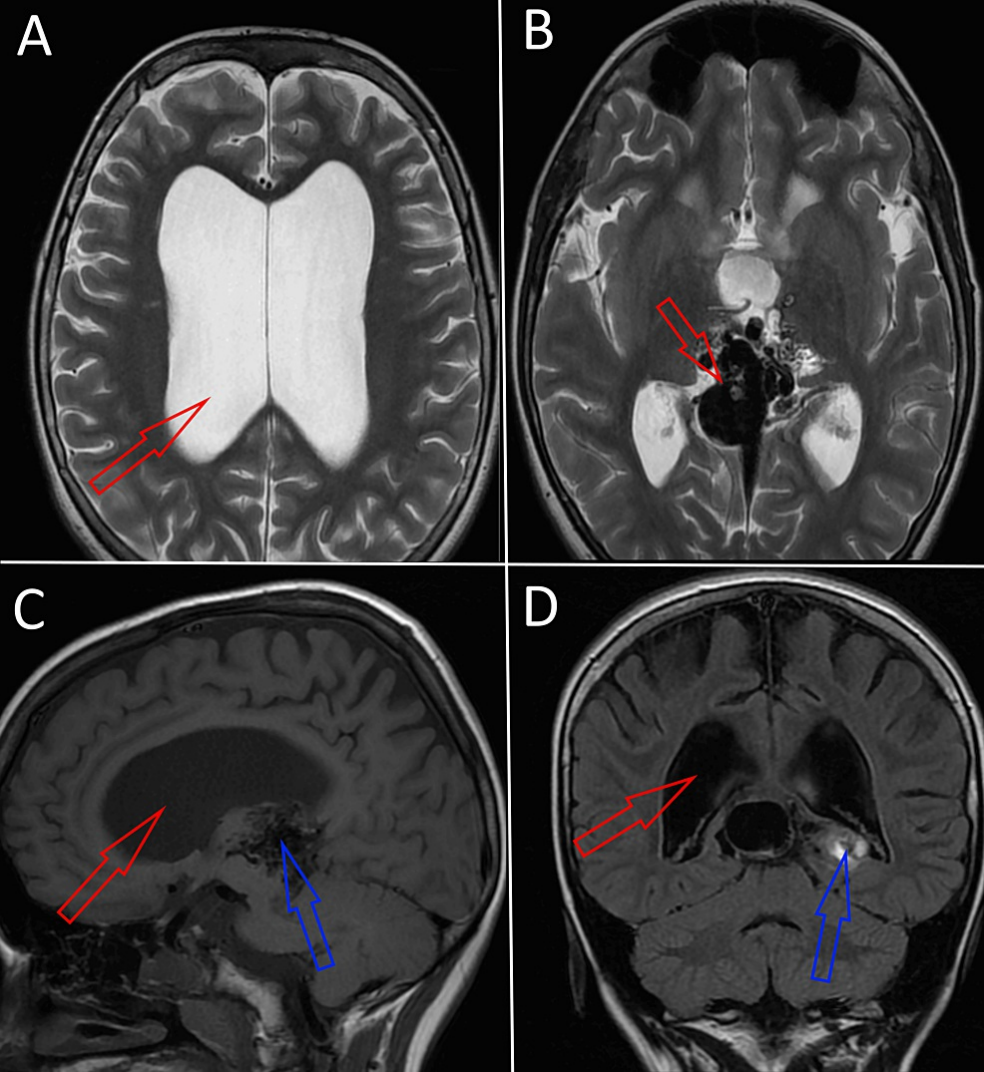
MRI taken two days after birth: (A) T2-weighted image from the axial plane showing abnormally enlarged lateral ventricles due to obstructive hydrocephalus; (B) T2-weighted image from the axial plane at the level of the prosencephalon, revealing the presence of a VGAM; (C) T1-weighted image from the sagittal plane revealing enlarged ventricles (red) and the presence of a VGAM (blue); (D) T1-weighted image from the coronal plane revealing enlarged ventricles (red) and increased blood flow into the VGAM (blue). VGAM: vein of Galen aneurysmal malformation.

The clinical history of the patient was extensive. The patient was born with a high Bicêtre score of 19, indicating a slight subclinical function of his vital systems, with no significant clinical manifestations. The obstructive hydrocephalus was diagnosed upon birth based on the patient’s abnormally large cranium and later confirmed via MRI (Figure 1). The same MRI revealed the presence of an aneurysmal malformation of a relatively large size in the median region of the prosencephalon (Figures 1B-1D).

**Figure 2:**
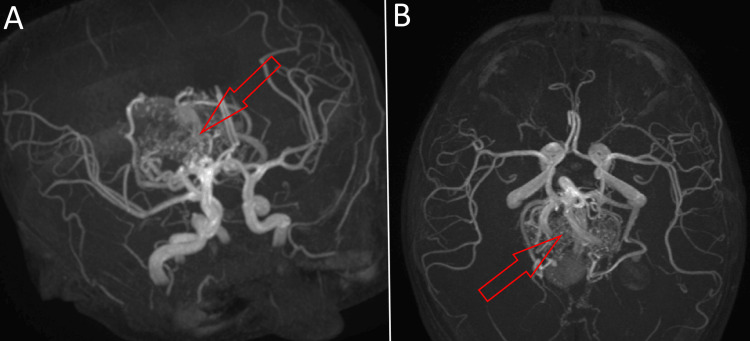
Contrast-enhanced MR-angiography of the patient's cerebral circulation shows the circle of Willis with the dilated median prosencephalic vein of Markowski clearly visible, alongside multiple arterial feeders: (A) inferolateral view; (B) axial view.

An appointed magnetic resonance angiography confirmed that the location of the malformation was the median prosencephalic vein of Markowski and revealed the type of the fistula to be choroidal, with a network of multiple posterior choroidal and thalamic arteries draining directly into the anterior segment of the vein and dilating it (Figure 2). Thus, the malformation confirmed the hydrocephalus as a secondary condition.

The patient’s parents opted for the hydrocephalus to be treated first by a pediatric neurosurgeon. An endoscopic third ventriculostomy was chosen as the treatment approach due to its better long-term prognosis and the nature of the obstruction. The intervention was performed at the age of four months under general anesthesia and was successful, with a lack of post-operative complications. Over the course of the following month, regular check-ups registered the presence of a short-term post-operative fever, which was treated with acetaminophen 15 mg orally and resolved after the first week. The neurosurgeon consulting the patient’s parents emphasized the importance of treating the VGAM in the near future, but the parents decided not to have it embolized at that point due to financial concerns.

**Figure 3:**
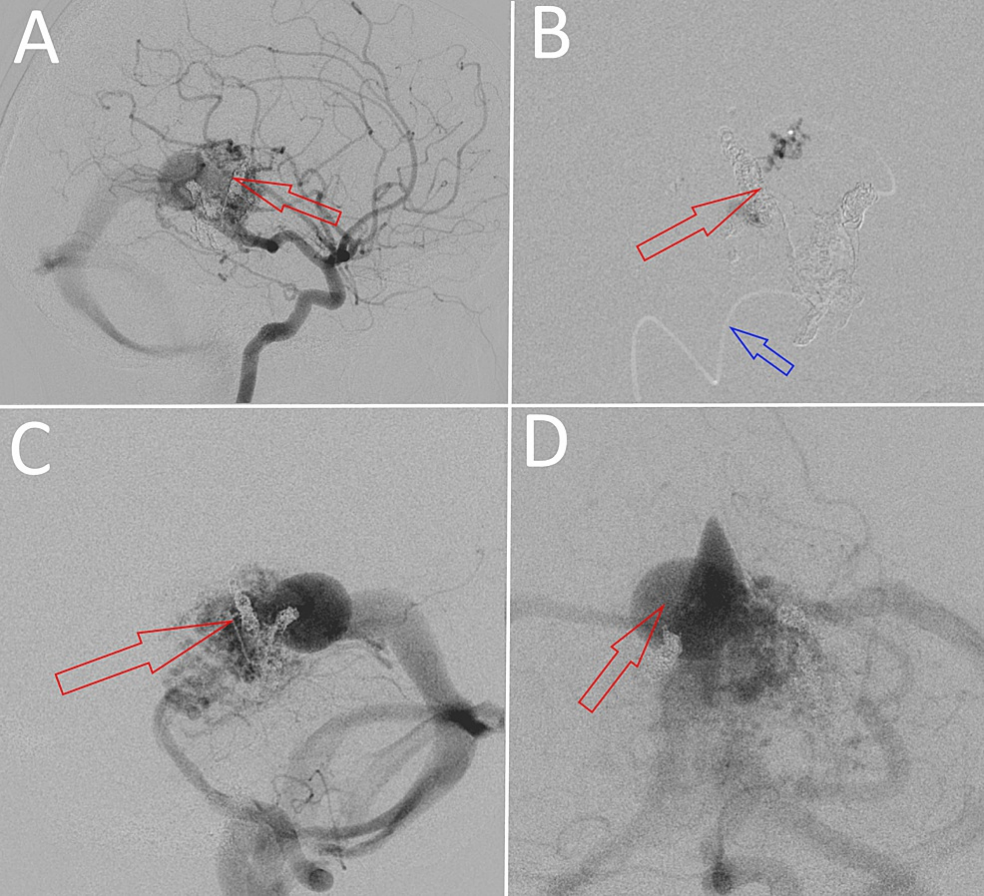
Post-embolization digital subtraction cerebral angiography showing subtotal occlusion of the arterial feeders of the malformation: (A) anterior cerebral artery-lateral view; (B) superselective contrast injection in a left-side arterial feeder vessel (blue) alongside obliterated fistulae (red); (C) internal carotid artery-lateral view; (D) internal carotid artery-frontal view.

When the patient was four years old (with normal intellectual, neurological, and physiological development), the VGAM was treated via a partial endovascular transarterial embolization with Onyx. A satisfying result was achieved, with most of the feeders, especially the posterior choroidal ones, being obliterated completely on the post-operative angiography (Figure 3). There were no intra- or post-operative complications to be mentioned, and a satisfying neurological outcome was reported at the six-month follow-up. Eight months after the embolization, the seizures started occurring. The patient was diagnosed with epilepsy after a consultation with a neurologist and treated for the condition with 150 mg of valproic acid orally daily. We concluded that the epilepsy was idiopathic and not a complication of the VGAM embolization procedure, as we could not establish a clear correlation between the pathology and the preceding operation.

For a period of one-and-a-half years, the patient’s condition was satisfactorily managed. Three weeks prior to the ER visit, the parents decided to lower the dosage of valproate (which at this point had reached 200 mg daily orally in accordance with the patient’s weight increase) to 100 mg orally daily because of the patient’s complaints of somnolence. Shortly thereafter, the severe seizure that brought the patient to the ER occurred. At the ER, there were suspicions that the status epilepticus was caused by an ischemic stroke. That possibility was dismissed upon the parents’ mention of the lowered valproic acid dosage and the absence of clinical manifestations characterizing cerebral ischemia, such as dysarthria, muscle weakness, and neurological deficits. The patient exhibited signs of being in a postictal state. He was dizzy, disoriented, and complained of a headache, but after half an hour, he returned to a regular state of consciousness with normal reflexes, awareness, and a satisfactory level of motor coordination. After administering a dose of 200 mg of valproic acid orally, a neurologist emphasized the importance of the patient being treated with the appropriate dose of medication to the parents. Afterward, the patient was discharged from the ER with a recommendation to be regularly monitored for possible long-term complications of his previous interventions.

## Discussion

The vein of Galen aneurysmal malformations is a rare type of congenital arteriovenous fistula developing between the sixth and 11th gestational week, in which one or multiple posterior choroidal or thalamic arteries shunt blood directly into the prosencephalic vein of Markowski, the embryonic precursor of the great cerebral vein (i.e., the vein of Galen), and thus pathologically dilate it [1,2]. The incidence of the lesion is estimated to be between one in 10,000 and 25,000 births, forming up to 30% of all reported congenital vascular anomalies in pediatric patients [2]. If a VGAM becomes symptomatic in the neonatal period and is left untreated, the outcome is almost always fatal and associated with multiple clinical complications, such as congestive heart failure (CHF), irreversible damage to the cerebral parenchyma causing multiorgan failure, cerebral hemorrhages, and hydrocephalus [1-3]. Diagnosis of the abnormality can be either antenatal via fetal MRI, 2D ultrasonography, Doppler or MR-angiography, or neonatal through the use of MRI, angiography, CT scan, or transfontanellar ultrasound, with MRI (both pre- and post-natal) being the most frequently used method [1,4]. However, there is no clear correlation between earlier prenatal diagnosis and better clinical outcomes in the long term, although antenatal imaging yields some benefits when it comes to pregnancy and delivery management of fetuses with VGAM [3,5]. Differential diagnoses of the lesion include cavum vergae and various intracranial cysts, among which cystic masses of the subarachnoid matter, pineal gland, the choroid plexus, cerebral parenchyma, and the pineal gland; however, all of these pathologies exhibit no blood flow with color Doppler [2].

For the purpose of planning out an appropriate treatment and prognosis, several classifications based on the lesion’s angioarchitecture are proposed, the most widely used of which is described by Lasjaunias; it differentiates the malformation as either mural (in which single or multiple arteriovenous fistulae shunt into the inferolateral margin of the wall of the prosencephalic vein of Markowski) or choroidal (in which multiple posterior choroidal and thalamic feeders shunt into the anterior segment of the vein) [3,5]. The choroidal type is associated with higher fatality rates and clinical manifestations such as severe macrocephaly, dilated orbital veins, and CHF early into the neonatal period due to the high flow of the fistulas, while the mural type frequently becomes symptomatic later in an individual’s life and rarely leads to CHF due to more restricted blood flow [1,4,5]. It should be noted that a VGAM is different from a vein of Galen aneurysmal dilatation (VGAD), in which there is an arteriovenous fistula draining into the true great cerebral vein rather than its embryonic precursor [5].

Treatment of VGAM can be carried out via surgery, medication, or endovascular embolization [5-7]. Surgery is associated with higher mortality rates and more significant complication risks for patients and often cannot be performed in neonates; therefore, it is not recommended or widely practiced, although it has been shown to produce some satisfying results in older infants and children with a mural type of malformation [5,7]. A medication course with the purpose of managing the patient’s elevated systemic vascular pressure (SVF) via catecholamines and inodilators can be an appropriate option in cases without CHF and an urgent need for embolization [6]. Endovascular embolization is regarded as the safest and most appropriate option for treatment in neonates and infants diagnosed with VGAM, especially when the lesion is choroidal, although there are significant perioperative and post-operative complications to be taken into consideration when performing the procedure (most commonly cerebral hemorrhages or hematomas, cerebral ischemia, hydrocephalus, and overall poor neurological outcomes) [1,3,5,7]. The embolization itself can be achieved via a transarterial or transvenous approach, with the former one being preferred for carrying the lesser risk of complications; however, when the arterial feeders of a choroidal malformation are too numerous and transarterial access is obstructed, a transvenous embolization may be the only viable option [1,3]. The complete occlusion of the lesion in some studies is associated with a better clinical outcome, although others redirect focus towards achieving a satisfying physiological and neurological outcome through partial embolization rather than striving for complete obliteration of the fistulae [3,8]. In some cases, a single embolization is insufficient and additional procedures are necessary [7]. The initial state of the patient is also an important factor to take into consideration. Neonates and infants with low Bicêtre scores, indicating multiorgan insufficiency, generally have a poor prognosis [8].

Hydrocephalus is the most common complication of VGAM as per the clinical literature and can arise during pre- or post-natal development [1-3,9]. The pathology can form due to hydrodynamic disorders associated with VGAM or mechanical obstruction of the aqueduct of Sylvius by the vascular mass [9]. While in some cases endovascular embolization by itself resolves the VGAM and the hydrocephalus secondary to it, in others (due to clinical or financial concerns regarding the embolization procedure), a complementary treatment method is necessary. The options in this case are a VP shunt or ETV. ETV is the recommended approach, as it is associated with considerably lower rates of complications in neonates and infants, while being a relatively safe and widely practiced procedure having a permanent morbidity rate of 2.38%, with the most common complications including post-operative fever (65-84%), intraoperative bradycardia (28-43%) and minor intracranial hemorrhages (16%) [9,10]. For comparison, a VP shunt in pediatric patients is associated with much more severe and frequent complications (such as irreversible parenchymal damage to the brain, shunt infections, shunt malfunctions, and abnormal ventricles) [10,11]. Park reports that, judging by the ETV success score (ETVSS) system described by Kulkarni et al., patients with an ETVSS above 80 have a very good initial and long-term prognosis, while those with moderate scores (50-70) have a moderate risk of initial ETV failure but a lower failure chance than a VP shunt in the long term (>three months); ETV is not recommended in cases with an ETVSS<40 [11]. We were unable to find research on whether ETV should be performed pre- or post-embolization, in which case it yields more satisfactory clinical outcomes. Guil-Ibáñez et al. describe several case studies in which ETV is performed pre- and post-embolization, with all the patients showing improvement in their condition regardless of the order of treatment procedures [9].

Epilepsy is a common complication associated with intracranial arteriovenous malformations and cerebral cavernous malformations (CCMs), with an incidence of up to 50%; however, it is not observed as a comorbidity or post-operative complication in VGAM and endovascular embolization treatment for the lesion [12]. We have found a single case report by Sayadnasiri describing VGAM-related epilepsy in an adult patient with an untreated, low-flow VGAM [13], but were unable to find any case reports of the complication arising in post-operative cases of pediatric patients.

## Conclusions

VGAM is a rare congenital abnormality that can lead to poor or fatal outcomes if not treated properly. In neonates and infants, it is typically treated through transarterial endovascular embolization when feasible. In cases where obstructive hydrocephalus is secondary to VGAM and primary pathology cannot be treated, an ETV can provide satisfactory results. Epilepsy is not associated with VGAM or its treatment, with limited evidence in rare cases. A pediatric patient with VGAM and secondary obstructive hydrocephalus was treated first for secondary pathology via ETV and later for transarterial endovascular embolization. The patient survived to four years with normal development before treatment, highlighting the need for further research on VGAM-related or embolization-related epilepsy.
